# Elucidation of Characteristic Sulfur-Fumigated Markers and Chemical Transformation Mechanism for Quality Control of *Achyranthes bidentate* Blume Using Metabolome and Sulfur Dioxide Residue Analysis

**DOI:** 10.3389/fpls.2018.00790

**Published:** 2018-06-12

**Authors:** Chuanzhi Kang, Dan Zhao, Liping Kang, Sheng Wang, Chaogeng Lv, Li Zhou, Jing-Yi Jiang, Wanzhen Yang, Jiaxing Li, Lu-Qi Huang, Lanping Guo

**Affiliations:** ^1^National Resource Center for Chinese Materia Medica, State Key Laboratory Breeding Base of Dao-di Herbs, China Academy of Chinese Medical Sciences, Beijing, China; ^2^Guiyang University of Chinese Medicine, Guiyang, China

**Keywords:** sulfur-fumigation, *Achyranthes bidentata* Blume, chemical transformation, characteristic markers, multivariate statistical analysis

## Abstract

*Achyranthes bidentata* Blume (AB) is a health food and a sulfur-free herbal medicine that is one of the most heavily sulfur-fumigated herbs in the marketplace. In this work, a comprehensive approach using ultra-performance liquid chromatography coupled with quadrupole time-of-flight-MS (UPLC-Q-TOF-MS) and multivariate statistical analysis was developed to identify characteristic sulfur-fumigation markers, elucidate chemical transformation mechanisms and characterize the degree of sulfur-fumigation of AB. Non-fumigated and sulfur-fumigated AB samples were compared by UPLC-Q-TOF-MS/MS analysis. Three triterpene saponins (Betavulgarosides II–IV) and two amides (Feruloyl-4-*O*-methyldopamine and Moupinamide) were identified as characteristic markers, which were positively correlated with two active AB components, namely oleanic acid and ferulic acid, respectively. Moreover, the extent of the sulfur-fumigation under different weight ratios of sulfur to herbal materials (1:20, 1:40, and 1:80) was analyzed based on chemical transformations and sulfur dioxide residues. Further verification showed that the ratio of 1:40 within 1 h was reasonable and efficient for herb quality preservation and assurance. This study provides a reliable sulfur-fumigation protocol for the quality control of AB and other herbs.

## Introduction

Traditionally, herbal materials have been handled during post-harvest by direct sun-drying or drying in the shade to obtain the dried root (China Pharmacopoeia [Part L], 2015). An alternative to sun-drying, sulfur-fumigation, has been widely employed to protect herbs from insects and molds, maintain their moisture, and promote a better appearance during post-harvest handling and storage (Kan et al., 2011; Jiang et al., 2013). However, large amounts of sulfur dioxide produced by sulfur-fumigation could induce chemical transformations of active components in herbs, and generate some new sulfur-containing derivatives (Duan et al., 2016; Li et al., 2017; Sun et al., 2017), and even lead to pharmacological risks, such as chronic bronchitis, asthma and cardiovascular diseases (Li et al., 2014; Pei et al., 2015; Ma et al., 2017). Therefore, the sulfur-fumigation processing method has been officially banned in TCM (China Pharmacopoeia [Part L], 2015). Nevertheless, this method is still been used by farmers and pharmacologists excessively and illegally, to extend storage time and increase the weight of herbs, especially for *Gastrodia Rhizoma*, *Codonopsis Radix, Dioscoreae Rhizoma*, and other TCM herbs with high polysaccharide content (Jiang et al., 2013; Ma et al., 2014; Kang et al., 2017). Currently, some countries and regions of the Pharmacopeia, such as the China Pharmacopeia, the European Pharmacopeia, the United States Pharmacopeia, and the Korea Pharmacopeia, have developed standard limit values of sulfur dioxide residues (European Pharmacopoeia 8.0, 2014; Korean Pharmacopoeia XI, 2014; United States Pharmacopoeia, 2014; China Pharmacopoeia [Part L], 2015). Although this comprehensive indicator reflects the content of total sulfur dioxide or sulfurous acid, it also indirectly reflects the loss of active components and health risk associated with sulfur-containing derivatives (Bai et al., 2015). This indicator does not specifically evaluate the extent of the sulfur-fumigation of herbal medicines. Our previous studies on sulfur-containing components of *Gastrodia Rhizoma* developed a practical protocol to identify characteristic sulfur-fumigated markers and dissected chemical transformation mechanisms and the extent of sulfur-fumigation (Kang et al., 2017). However, few studies have investigated the relationship between the extent of sulfur-fumigation to herb quality preservation and assurance. It is our aim to further explore whether or not this practical protocol is reliable and applicable to all sulfur-fumigated herbs, especially those with no indigenous sulfur.

*Achyranthes bidentata* Blume (AB) commonly known as “Niuxi” in traditional Chinese medicines (TCM), is one of the most important herbal medicines and health foods (Yuan et al., 2010; China Pharmacopoeia [Part L], 2015; He et al., 2017). Henan province is considered to be the “Dao Di” area of AB, where it is known as “Huai Niuxi” (Yan S.-X. et al., 2016). As a result of AB has good medicinal value and pharmacological activity (Vetrichelvan and Jagadeesan, 2002; Shen et al., 2011; Liu et al., 2016) for invigorating livers and kidneys (Mitaine-Offer et al., 2001a,b; Li et al., 2005; Liu et al., 2016; Yang et al., 2017), and sulfur-fumigation has been widely employed to protect AB from insects and molds, AB has become excessively sulfur-fumigated in China. And sulfur-fumigation could cause significant alteration of active constituents in AB. Therefore, AB is selected as a model herb for rationally monitoring sulfur-fumigation. Furthermore, many chemical indicators have been studied to evaluate the quality of AB, but indicators for sulfur-fumigated AB still remain unknown.

In the present study, we analyzed the metabolome using ultra-performance liquid chromatography coupled with quadrupole time-of-flight-MS (UPLC-Q-TOF-MS) (Li et al., 2012; Ma et al., 2014) and ultra-performance liquid chromatography tandem mass spectrometry (UPLC-MS/MS) and performed multivariate statistical analyses (Yan Y. et al., 2016), to rationally and dynamically monitor characteristic metabolites and dissect chemical transformation mechanisms during AB sulfur-fumigation. In addition, the different weight ratios of the sulfur to herb material were determined during multiple time points during the process of sulfur-fumigation (Kang et al., 2017) to investigate the extent of the sulfur-fumigation, with the aim of providing a scientific and practical protocol for evaluating and controlling the quality of sulfur-fumigated AB and other herbal materials.

## Materials and Methods

### Chemicals, Reagents, and Herbal Materials

HPLC grade acetonitrile was obtained from Merck Co. (Darmstadt, Germany). MS grade formic acid and analytic grade methanol were obtained from Fisher Scientific (Geel, Belgium). Ultra-pure water was produced by a Thermo Scientific purifier system (Langenselbold, Germany). The reference compounds of ecdysone (EC), betaine (BE), oleanic acid (OA), ferulic acid (FA), and 5-hydroxymethyl furfural (5-HF) were purchased from Shanghai Shifeng Biological Technology Co., Ltd. (Shanghai, China). Their structures are shown in Supplementary Figure S1.

Fresh AB samples (10 kg) were collected from the “Dao Di” area of Jiaozuo (Henan, China) in January 2017. All the samples were identified as *Achyranthes bidentata* Blume by Prof. Lan-Ping Guo. The voucher specimens (No. 2017-215R) were deposited at the National Resource Center for Chinese Materia Medica, China Academy of Chinese Medical Sciences.

### Sulfur Fumigation of AB

The fresh AB samples were randomly separated into three groups, based on the weight ratio of sulfur to herbal material, specifically 1:20, 1:40, and 1:80. Subsequently, each group of samples were further separated into seven portions to study the extent of sulfur-fumigation at different times. Sulfur-fumigation of AB was performed as described previously (Kang et al., 2017). A sealed plastic installation separated into upper and lower layers was designed with two small holes in the side walls to simulate the sulfur-fumigation conditions used by herb farmers. The fresh AB samples were placed in the upper section and sulfur was placed in the lower section of the apparatus. The duration of fumigation was set to 1, 2, 4, 8, 12, and 24 h. All fumigated and non-fumigated samples were dried at 50°C. All the pulverized samples were prepared in three biological replicates and stored at 4°C for further analysis.

### Preparation of Sample Solutions and Standard Solutions

Fifty milligrams of the AB powder was accurately weighed and extracted by ultrasonication for 60 min (40 KHz) with 50% methanol at a concentration of 50 mg/mL to prepare the samples. The extracted solutions were centrifuged for 10 min at 13,000 rpm, and the supernatants were then filtered through 0.22 μm microporous filters for UPLC-Q-TOF/MS qualitative and UPLC-MS quantitative analysis. An internal standard, *p*-hydroxybenzoic acid, was used for correcting the mass spectral response and semi-quantitative analysis by UPLC-Q-TOF/MS.

Standard stock solutions of EC, BE, OA, FA, and 5-HF were accurately weighed and directly dissolved in 50% methanol (v/v) to a concentration of 1 mg/mL. Every standard solution was mixed and diluted with 50% methanol to a final concentration of 104.00 μg/mL for EC, 147.00 μg/mL for BE, 110.00 μg/mL for OA, 5.40 μg/mL for FA, and 7.80 μg/mL for 5-HF and stored at 4°C for further analysis.

### Sulfur Dioxide Residue Analysis

The sulfur dioxide residue of all sulfur-fumigated AB samples were determined by iodine titration, a standard method documented in Chinese Pharmacopeia 2015 version (Part four) Appendix 2331 (China Pharmacopoeia [Part IV], 2015).

### UPLC-QTOF-MS/MS Analysis

#### UPLC/QTOF-MS^E^ Analysis

A Waters ACQUITY UPLC^®^ system (Waters, Milford, MA, United States) coupled with Xevo G2-S QTOF-MS (Waters Micromass, Manchester, United Kingdom) were used for the analysis of metabolites changes of AB before and after sulfur-fumigation. A Waters ACQUITY HSS T3 column (1.8 μm, 2.1 mm × 100 mm) with the column temperature set at 40°C was used to analyze the AB extracts. The gradient mobile phase was 0.1% (v/v) formic acid (A) and 0.1% (v/v) formic acid acetonitrile (B) with the injection volume set at 1 μL and a flow rate of 0.5 mL/min. The optimized UPLC elution conditions were 0–5% B (0–2 min), 5–25% B (2–6 min), 25–40% B (6–10 min), 40–70% B (10–16 min), 70–95% B (16–22 min), and 95% B (22–27 min).

Waters Xevo G2-S Q-TOF-MS equipped with an electrospray ionization (ESI) source was performed in positive ion mode. The parameters of ESI(–) were set as follows: capillary voltage, 2.0 kV; cone voltage, 40 V; dissolved gas temperature, 450°C; source temperature, 100°C; cone gas flow, 50 L/h; desolvation flow rate, 900 L/h; scan range, 50–1,500 Da; scan time, 0.2 s; collision energy (CE), 30–50 V.

#### Multivariate Statistical Analysis

Six samples (include three biological replicates and two replicates parallel to each biology) selected from non-fumigated and sulfur-fumigated groups, respectively, were used for multivariate statistical analysis. The raw data of twelve samples were acquired with the MassLynx^TM^ software (version 4.1, Waters Co., Milford, MA, United States) and further transferred into the Progenesis QI software (Waters Co., Milford, MA, United States) to dissect the potential characteristic compounds of sulfur-fumigated and non-fumigated AB on the basis of accurate mass, retention time, and abundance (Kang et al., 2017). Other parameters were as follows: retention time tolerance, 0.20 min; mass tolerance, 5.0 ppm. Subsequently, the algorithm of ANOVE *p*-value, minimum coefficient of variation (CV) and max fold change were used to filter compounds. The resulting data were exported to EZinfo software (version 2.0.0.0) for principal component analysis (PCA) and orthogonal partial least squared discriminant analysis (OPLS-DA) analysis.

### Determination of Five Major Active Components

### UPLC-MS/MS Analysis

A Waters ACQUITY UPLC^®^ system (Waters, Milford, MA, United States) coupled with AB SCIEX QTRAP^®^ 6500 (AB SCIEX, Redwood City, CA, United States) mass spectrometer with ESI ionization in multiple reaction monitoring (MRM) detection mode were used for quantitative analysis of AB samples. The chromatographic Waters ACQUITY HSS T3 column (1.8 μm, 2.1 mm × 100 mm) was used with the column temperatures set at 35°C. The mobile phase consisted of 0.1% (v/v) formic acid solution (A) and acetonitrile (B) with a flow rate of 0.5 mL/min. The optimized gradient program was as follows: 5–15% B (0–1 min); 15–40% B (1–2.5 min); 40–90% B (2.5–4 min); 90–95% B (4–6.5 min); 95% A (6.5–8 min); 95–5% B (8–8.5 min); 5% B (8.5–10 min). The injection volume was set as 1 μL. QTRAP^®^ 6500 MS equipped with ESI source was performed both in positive and negative ion modes with a source temperature of 550°C. The curtain gas (CUR) was set to 35 psi. The detailed optimized parameters of MS and MS/MS analysis including declustering potential (DP), CE, entrance potential (EP), etc. are shown in Supplementary Table S1.

#### Method Validation

The quantitative analysis method was fully validated for specificity, linearity, limit of detection (LOD), limit of quantitation (LOQ), intra-day and inter-day precision, accuracy, and stability. Specifically, the standard stock solutions were diluted to a series of appropriate concentrations, and then the LOD and LOQ were determined at a signal-to-noise ratio (S/N) of about S/N = 3 and S/N = 10, respectively. The accuracy test was set at low (80% of the original amount), medium (100% of the original amount) and high (120% of the original amount) levels (Kong et al., 2014).

#### Statistical Analysis

The quantitative analysis was performed by Analyst^®^ software (version 1.6.2) and MultiQuant^TM^ software (version 3.0). The results from 84 AB samples were expressed as mean values ± standard deviations of triplicates. A one-way analysis of variance (ANOVA) was performed using GraphPad Prism 5 (GraphPad Software, Inc., Version 5.01). The resulting data were exported to SIMCA software (MKS UMETRICS AB, version 14.1.0) for PCA and partial least squared discriminant analysis (PLS-DA).

## Results and Discussion

### Comparison of Metabolites by UPLC-Q-TOF-MS

#### Optimization of Chromatographic and Mass Spectrometric Conditions

To obtain satisfactory extraction efficiency and response from the fragment ions, various types of extraction solvents (ethanol, 50% methanol, 80% methanol, and 100% methanol) and different extraction times (30, 60, and 90 min) were evaluated. A concentration of 50% methanol and a 60 min extraction time were selected for further analysis (Supplementary Figure S2). Additionally, parameters such as source temperature, dissolved gas temperature, capillary voltage, cone voltage, cone gas flow, desolvation flow rate and CE were also optimized to obtain precise molecular weights and the highest intensity of fragment ions (Lai et al., 2016). Moreover, the positive mode was chosen for its higher peak capacity and better resolution of the major components of AB (Supplementary Figure S3).

#### UPLC-Q-TOF-MS-Based Metabolome for Exploring the Marker Metabolites

**FIGURE 1:**
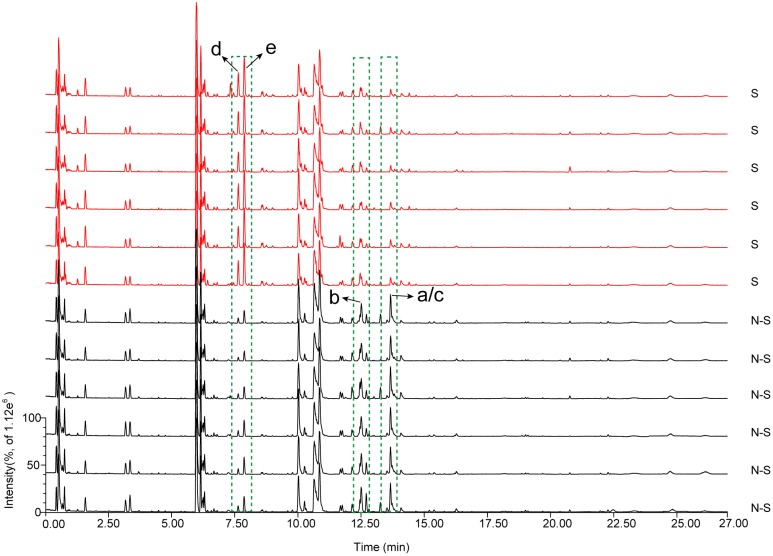
Overlay of total ion chromatograms of AB extracts for non-fumigated (N-S) and sulfur-fumigated (S) in positive mode (compounds a–e are the significantly different compounds).

To analyze the characteristic metabolites of AB before and after sulfur-fumigation, six samples (include three biological replicates, and two replicates parallel to each biology) selected from the non-fumigated (0 h) and the sulfur-fumigated (2 h) treatments in the most heavily fumigated group (1:20) were analyzed by TOF-MS, respectively, (Supplementary Figure S4 and **Figure 1**). Some components of AB were obviously changed after sulfur-fumigation, such as unknown components a–e, indicating that the chemical composition of AB with respect to sulfur dioxide or sulfite had undergone some redox or degradation reactions (Duan et al., 2016). Subsequently, the mass spectrometry data obtained from MassLynx software were exported to Progenesis QI software for peak shape aligning and ion screening analysis (Lin et al., 2015). The parameters and their settings were as follows: the minimum coefficient of variation (value ≥ 2), ANOVA *p*-value (*p* ≤ 0.05) and max fold change (value ≥ 2). Subsequently, 350 ions representing chemical differences were selected from 2173 aligning ions, and then imported to EZinfo software for PCA and OPLS-DA analysis.

**FIGURE 2:**
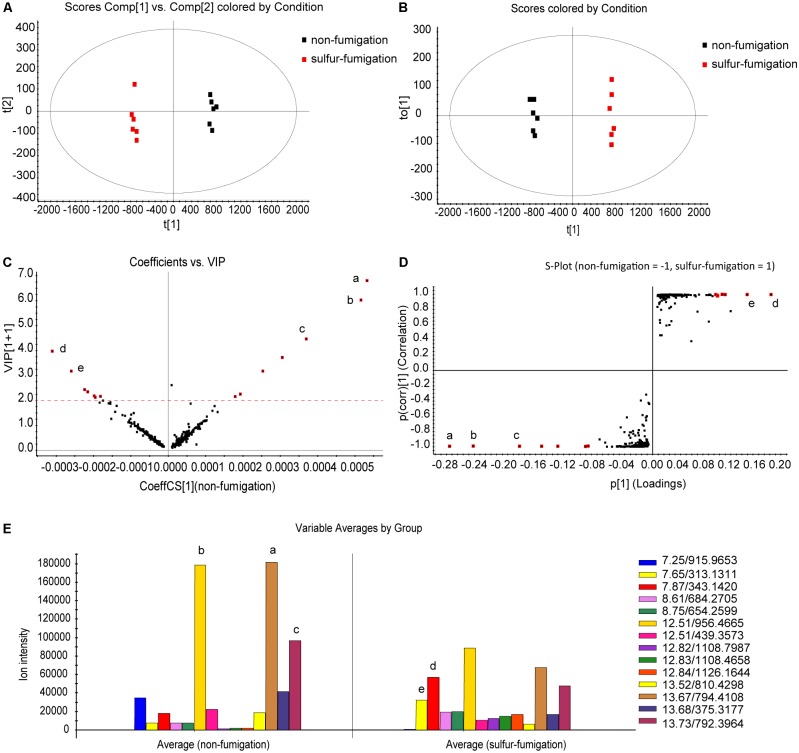
Multivariate statistical analysis of methanol extracts of non-fumigated GR (0 h) and sulfur-fumigated AB (2 h) samples. **(A)** PCA/scores plot (six samples with each group, three biological replicates); **(B)** OPLS-DA/scores plot (six samples with each group, three biological replicates); **(C)** VIP plot; **(D)** S-plot; **(E)** column plot of the ion intensity (compounds a–e are significantly different markers in AB samples).

The scale type was set to Par scaling with mean-centring before data pre-processing (Lin et al., 2015). The analysis revealed that non-fumigated and sulfur-fumigated samples were clearly clustered into two groups in PCA and OPLS-DA plots (**Figures 2A,B**). To further screen for potential marker metabolites that can discriminate between non-fumigated and sulfur-fumigated AB samples, the indicators of variable importance in the projection (VIP) values (VIP > 2.0) and the S-Plot were used. Finally, seven distinctive marker ions in non-fumigated and sulfur-fumigated AB were selected (**Figures 2C,D**). Subsequently, five marker ions from all fourteen marker ions including **a** (t_R_ 13.67 min, *m/z* 794.4108), **b** (t_R_ 12.51 min, *m/z* 956.4665), **c** (t_R_ 13.73 min, *m/z* 792.3964), **d** (t_R_ 7.87 min, *m/z* 343.1420), and **e** (t_R_ 7.65 min, *m/z* 313.1311) were selected according to the ion intensity (**Figure 2E**), i.e., the ions **a**, **b**, and **c** were detected with higher intensity in non-fumigated compared to sulfur-fumigated AB, while the ions **d** and **e** were detected with higher intensity in sulfur-fumigated compared to non-fumigated AB.

**FIGURE 3:**
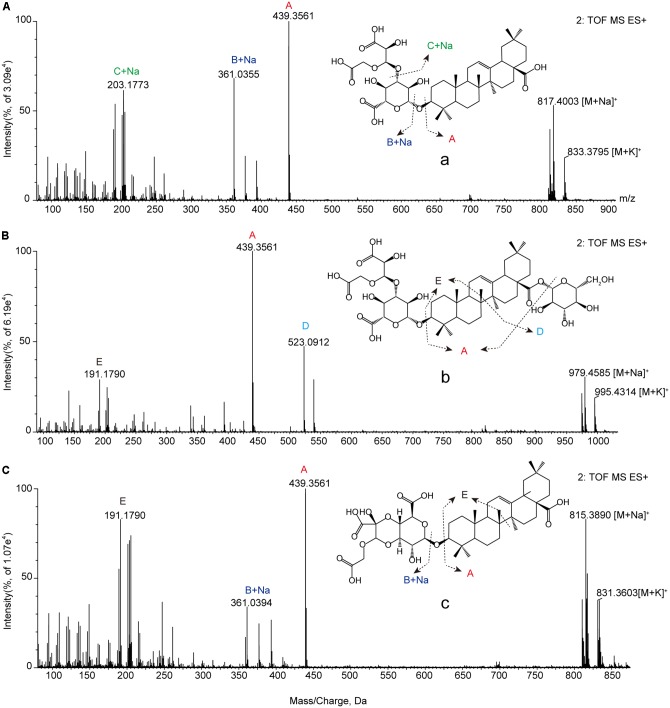
The positive MS/MS spectrum of characteristic chemical markers. **(A)** Marker a (Betavulgaroside IV); **(B)** marker b (Betavulgaroside III); **(C)** marker c (Betavulgaroside II).

**FIGURE 4:**
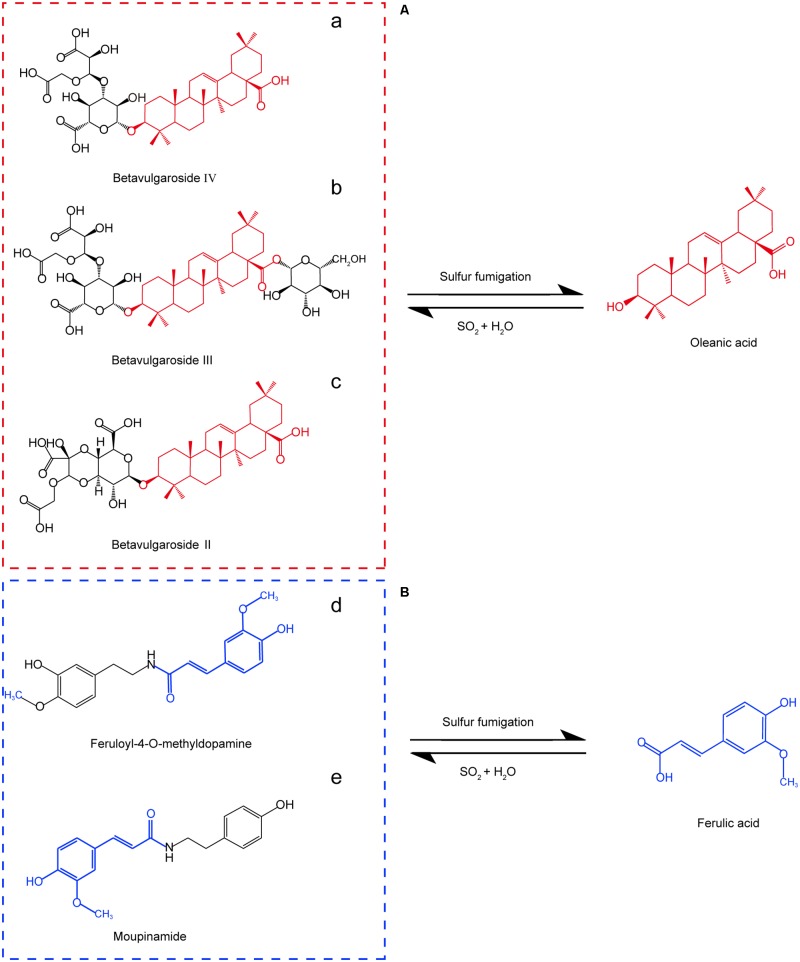
Five characteristic chemical markers and transformation of sulfur-fumigated AB. **(A)** The intensity of three markers (a, b. and c) declined after sulfur-fumigation; **(B)** the intensity of two markers (d and e) increased after sulfur-fumigation.

We used retention times and fragmentation patterns of marker ions (Li et al., 2015; Lai et al., 2016), to explain fragmentation regularities and chemical structures. For marker **a**, the fragment ion at *m/z* 795.4108 was detected at 13.67 min and identified as quasi-molecular [M+H]^+^, with the possible elemental composition of C_41_H_61_O_15_. The adduct ions at *m/z* 817.4003 [M+Na]^+^ and 833.3795 [M+K]^+^ were also detected. Furthermore, this marker showed three high energy MS ions including A (*m/z* 439.3561), B (*m/z* 361.0355), and C (*m/z* 203.1773) (**Figure 3A**). Consequently, marker **a** was inferred to be Betavulgaroside IV (C_41_H_62_O_15_). The other specific fragmentation patterns of marker ions in non-fumigated and sulfur-fumigated AB are shown in **Figure 3** and Supplementary Figure S5. Ultimately, the transformation mechanism of the five markers resulted in the identification of three triterpene saponins (Betavulgarosides II–IV) in non-fumigated and two amides (Feruloyl-4-*O*-methyldopamine and Moupinamide) in sulfur-fumigated AB (**Figure 4**). The identities of these five markers were consistent with the five characteristic difference metabolites in **Figure 1**. To determine the relative contents of the five markers in AB before and after sulfur-fumigation, *p*-hydroxybenzoic acid (0.001 mg/mL) was chosen as an internal standard. The relative contents of **a** (1.9037 mg/g), **b** (1.8662 mg/g), and **c** (1.0132 mg/g) in non-fumigated samples corresponded to 0.5265 mg/g, 0.7155 mg/g, and 0.3741 mg/g in sulfur-fumigated AB, respectively, which was very significantly different (*p* < 0.001), especially for marker **a** (down 3.6-fold) (Supplementary Figure S6). We also done the hydrolytic reactions using ginsenoside Ro under sulfur fumigation, and verified the transformation mechanism of the three triterpene saponin components through hydrolytic reactions (Supplementary Figure S7). In general, the contents of the five characteristic chemical markers can indirectly reflect the degree of sulfur fumigation on the quality of AB. In addition to the five identified markers, nine other unidentified markers (including two ion fragments) are also shown in **Table 1**.

**Table 1:** Fourteen marker ions of non-fumigated and sulfur-fumigated AB samples in positive mode.

Group	No.	RT	*m/z*	Cal. *m/z*	Factor of change	Mass accuracy (ppm)	Formula	Name	MS/MS fragment ion (*m/z*)
Non-fumigation	1	7.25	915.9653	915.9658	16.44^b^	-0.7	C_27_H_17_NO_35_	–	373.1383, 245.1129, 226.0824, 136.0744, 86.0951
	2^a^	12.51	439.3573	439.3576	20.11	-3.4	C_30_H_46_O_2_	–	393.3511, 288.2879, 147.9303
	3	12.51	974.4951	974.4961	82.01	-1.0	C_47_H_72_O_20_	Betavulgaroside III	979.4585[M+Na]^+^, 974.5014[M+NH_4_]^+^, 523.0912, 439.3561, 393.3511, 339.0544, 203.1773, 191.1790, 141.0167
	4	13.52	810.4298	810.4275	30.07	2.6	C_41_H_63_NO_15_	–	831.3662, 439.3561, 377.0302, 247.1708, 203.1773, 191.1790, 149.1299, 95.0848
	5	13.67	812.4447	812.4432	122.70	1.8	C_41_H_62_O_15_	Betavulgaroside IV	833.3795[M+K]^+^, 812.4432[M+NH_4_]^+^, 817.4003[M+Na]^+^, 439.3561, 361.0355, 247.1708, 203.1773, 191.1790, 149.1299, 95.0848
	6^a^	13.68	439.3561	439.3576	20.51	-3.4	C_30_H_46_O_2_	–	393.3511[M+NH_4_]^+^, 361.0355, 203.1773, 191.1762, 149.1299
	7	13.73	810.4297	810.4275	52.04	2.6	C_41_H_63_NO_15_	Betavulgaroside II	831.3603[M+K]^+^, 810.4302[M+NH_4_]^+^, 439.3561, 361.0394, 247.1676, 191.1790, 149.1324
Sulfur-fumigation	8	7.65	314.1407	314.1392	40.23^c^	4.8	C_18_H_19_NO_4_	Moupinamide	352.0980[M+K]^+^, 314.1407, 177.0539, 145.0277, 121.0637, 103.0532, 89.0382
	9	7.87	344.1490	344.1498	32.20	-2.6	C_19_H_21_NO_5_	Feruloyl-4-*O*-methyldopamine	382.1053[M+K]^+^, 344.1490[M+H]^+^, 177.0539, 145.0277, 117.0315, 89.0363
	10	8.61	685.2760	685.2761	20.57	-0.1	C_38_H_40_N_2_O_10_	–	723.2376[M+K]^+^, 707.2628[M+NH4]^+^, 520.1982, 383.1140, 351.0850, 263.0702, 231.0634, 201.0506, 121.0637
	11	8.75	655.2653	655.2655	12.56	-0.5	C_37_H_38_N_2_O_9_	–	677.2429[M+Na]^+^, 490.1854, 353.1048, 321.0734, 231.0665, 201.0506, 121.0637
	12	12.82	1126.8279	1126.8286	9.87	-0.6	C_70_H_111_NO_10_	–	965.4451, 949.4571, 493.0893, 439.3561, 374.2103, 203.1773, 191.1818, 141.0192
	13	12.83	1126.4997	1126.4974	9.20	0.5	C_43_H_83_NO_32_	–	965.4324, 493.0802, 374.2143, 233.1334, 203.0169, 191.1790, 141.0167, 121.0975, 86.0951
	14	12.84	1126.1644	1126.1641	9.01	0.1	C_41_H_43_NO_36_	–	949.4571, 523.0959, 493.0711, 439.3561, 205.1920, 141.0167, 86.0951

*-, not identified; ^a^ion fragment; ^b^the markers higher level in non-fumigation than in sulfur-fumigation; ^c^the markers higher level in sulfur-fumigation than in non-fumigation.*

### Dynamics of Five Major Active Components of AB During the Sulfur-Fumigation Process Using UPLC-MS/MS

#### Optimization of Chromatographic Parameters and Sample Preparation

As shown in **Figure 4**, the contents of OA and FA can reflect the dynamics of the five markers (three triterpene saponins and two amides) during the sulfur-fumigation process. Moreover, the reference compounds of these five markers were difficult to obtain. Therefore, five major components (EC, OA, BE, FA, and 5-HF) of AB were chosen to indirectly evaluate the overall sulfur-fumigation process. The main purpose is to explain the trend of changes in the degree of sulfur-fumigation. Before quantitative analysis, extraction solvents and extraction times were first evaluated at 250 nm wavelength to obtain satisfactory extraction efficiency. As mentioned before, the best extraction method was 50% methanol with ultrasonication for 60 min (Supplementary Figure S8). The mass spectrometric conditions of five major components were also optimized, and the specific parameters such as DP, CE, and CXP are shown in Supplementary Table S1. The optimized chromatogram of the five components are shown in Supplementary Figure S9.

#### Method Validation

The specificities were validated by comparing the retention time and peak shape of each analyte in AB samples with the reference standard and showed that the five analytes could all be identified with good resolution by ion pairs (Supplementary Figure S9). The calibration curves of each analyte showed good linearity (*R*^2^ > 0.9988). The LOD and LOQ were calculated to be 0.0014–0.0055 μg/mL and 0.0048–0.0177 μg/mL, respectively. Precision was evaluated by intra-day and inter-day variability, and the result showed the RSDs were less than 3.43%. The repeatability was analyzed by six test solutions of the same AB samples, and the RSDs ranged from 1.87 to 2.80%. The stability was tested by analysing the peak areas of sample extracts stored at 25°C for 0, 2, 4, 8, 12, and 24 h. The RSDs for the five analytes were less than 3.16%. All the method validation data are summarized in **Table 2**. Moreover, the recovery of the five analytes ranged from 93.0059 to 105.3923% with RSD < 3.24% (Supplementary Table S2). These results demonstrate that the UPLC-MS/MS method was precise, stable and accurate enough for simultaneous quantification of these five analytes in AB.

**Table 2:** The regression equation, LOD and LOQ of the eight standards in AB samples using the optimized method for calibration.

Analyte	Calibration curve	*R*^2^	Linear range (μg/mL)	LOD (μg/mL)	LOQ (μg/mL)	Repeatability (*n* = 6)	Stability (RSD %)	Intra-day (RSD%, *n* = 6)			Inter-day (RSD%, *n* = 3)		
								Low	Middle	High	Low	Middle	High
EC	*Y* = 32279 *X*+ 47361	0.9994	5.20 – 104.00	0.0015	0.0052	2.09	2.66	1.97	2.82	1.40	1.22	2.41	1.43
BE	*Y* = 223169*X*+ 1.91 × 10^7^	0.9988	7.35 – 294.00	0.0015	0.0050	2.80	2.19	1.41	0.94	1.27	1.51	0.91	1.66
OA	*Y* = 80780*X* + 4164.4	0.9993	82.50 – 825.00	0.0055	0.0177	1.87	3.16	1.67	2.04	2.62	0.60	1.37	2.76
FA	*Y* = 2.46 × 10^6^*X* – 2.57 × 10^3^	0.9998	0.01 – 5.40	0.0014	0.0048	2.67	2.51	3.43	1.01	1.39	2.70	2.44	1.73
5-HF	*Y* = 1.88 × 10^6^*X* + 6.56 × 10^3^	0.9996	0.013 – 7.80	0.0023	0.0077	2.06	2.00	0.97	1.18	1.28	1.70	2.06	1.98

#### Dynamic Monitoring of the Sulfur-Fumigation Process of AB Samples

**FIGURE 5:**
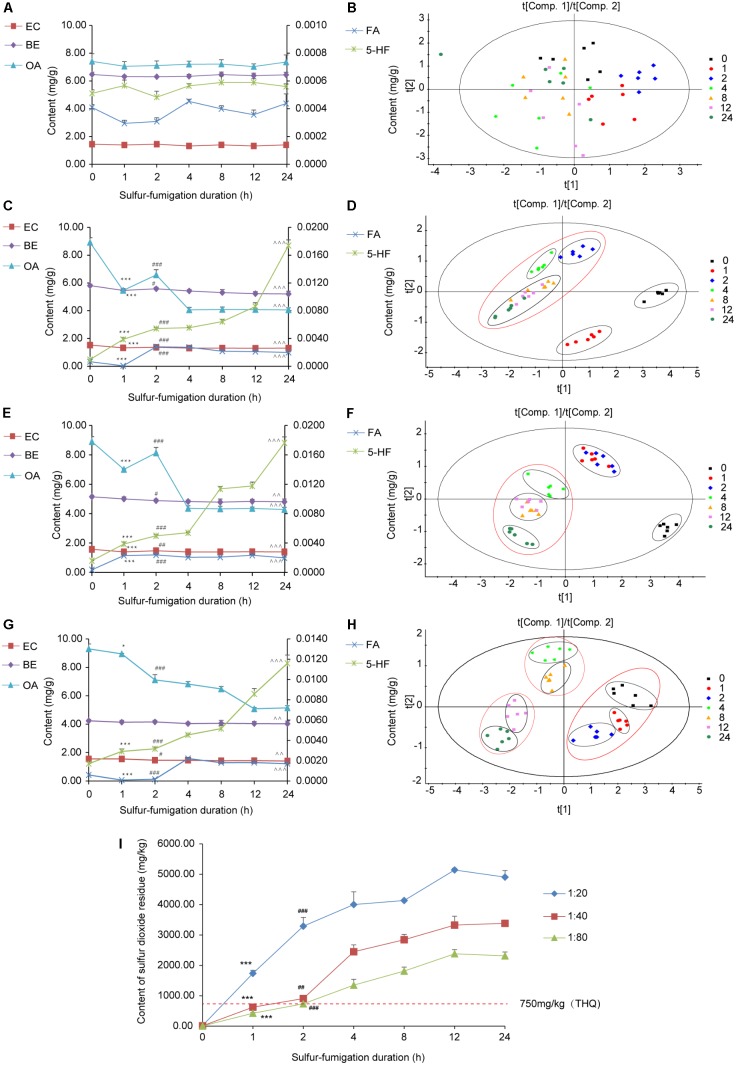
Content variations and PLS-DA coefficient plot of the five major compounds and sulfur dioxide residue in the AB sample within 24 h sulfur-fumigation. **(A)** Non-fumigated sample; **(B)** non-fumigated sample (PLS-DA); **(C)** the weight ratio of sulfur to AB (1:20); **(D)** the weight ratio of sulfur to GR (1:20) (PLS-DA); **(E)** the weight ratio of sulfur to AB (1:40); **(F)** the weight ratio of sulfur to GR (1:40) (PLS-DA); **(G)** the weight ratio of sulfur to AB (1:80); **(H)** the weight ratio of sulfur to GR (1:80) (PLS-DA). (^∗∗∗^*p* < 0.001, ^∗^*p* < 0.05, compared with the 0 h; ^###^*p* < 0.001, ^##^*p* < 0.01, ^#^*p* < 0.05, compared with the 0 h; ˆˆˆ*p* < 0.001, ˆˆ*p* < 0.001, compared with the 0 h) **(I)** content variations of sulfur dioxide residue in sulfur-fumigation process (^∗∗∗^*p* < 0.001, compared with 0 h; ^###^*p* < 0.001, ^##^*p* < 0.01 compared with 1 h). All samples include three biological replicates, and two replicates parallel to each biology.

To monitor the sulfur-fumigation process of AB and dissect the chemical transformation mechanisms of the screened markers, five major active AB compounds (EC, BE, OA, FA, and 5-HF) were simultaneously determined at seven time points within 24 h, as well as four weight ratios of sulfur to herbal materials (blank, 1:20, 1:40, and 1:80) (Supplementary Table S3). Furthermore, PCA and PLS-DA analysis with SIMCA software were used to further evaluate the extent of the sulfur-fumigation (**Figure 5**) (Li et al., 2016; Kang et al., 2017). Overall, the quality of AB was significantly different before and after sulfur-fumigation. The content change of the analytes in AB mainly occurred in the first 2 h and gradually weakened with the reduction of weight ratios of sulfur to herbal materials (**Figures 5C,E,G**). It can be inferred that hydrolysis, sulfitation, and their reverse reactions, which are related to the formation of sulfurous acid, organic acid and steroids, occurred simultaneously (Duan et al., 2016; Kang et al., 2017; Sun et al., 2017). Particularly, the three triterpene saponin markers (Betavulgarosides II–IV) were extremely relevant to OA, and the two amide markers (Feruloyl-4-*O*-methyldopamine and Moupinamide) were extremely relevant to FA, based on the chemical structures and the hydrolytic reactions of three triterpene (**Figure 4** and Supplementary Figure S7).

Specifically, as shown in **Figures 5A,B**, the contents of five analytes in unfumigated AB did not change significantly during storage of 24 h (*p* > 0.05, compared with 0 h). After 24 h of sulfur-fumigation, the contents of BE, EC, and OA all significantly declined (*p* < 0.001, compared with the 0 h), while the content of FA and 5-HF increased significantly (*p* < 0.001, compared with the 0 h), especially for the latter (**Figures 5C,E,G**). PLS-DA analysis showed that 1 h and 2 h were important time points to reflect the extent of the sulfur-fumigation, probably corresponding to the time when the sulfur dioxide permeated from the superficial tissues into the deeper tissues of the plant material. As shown in **Figures 5D,F,H**, based on the clustering observed after 1 h and 2 h of fumigation, the whole herb material could be permeated by sulfur dioxide after 2 h of sulfur-fumigation at the ratio of 1:20. However, 8 h of sulfur-fumigation at the ratio of 1:80 was sufficient for sulfur dioxide to permeate into the superficial tissues completely. On the other hand, at the ratio of 1:40, 1 h or 2 h of sulfur-fumigation was enough to permeate into the superficial tissues exactly; this could represent a better process to ensure the quality preservation of AB.

As shown in **Figure 5I**, the sulfur dioxide residue in AB was significantly and positively correlated with the amount of sulfur, and showed an increasing trend before 12 h of sulfur fumigation and then gradually became steady. After only 1 h of sulfur-fumigation, the sulfur dioxide residue from three groups (1:20, 1:40, and 1:80) increased quickly and exceeded the standard [400 mg/kg in Chinese Pharmacopeia (2015 edition)] by the amounts of 1,738.04 mg/kg, 625.10 mg/kg, and 425.10 mg/kg, respectively. Our previous study put forward the maximum residue limit (MRL) of sulfur dioxide intake from herbs (750 mg/kg) based on the target hazard quotient (THQ) and total daily food consumption (Kang et al., 2017). Therefore, combined with the extent of sulfur fumigation and the safety limit of sulfur dioxide, the weight ratio of sulfur to AB of 1:40 within 1 h was the best sulfur-fumigation protocol to ensure AB quality, safety and preservation.

## Conclusion

Along with our previous study (Kang et al., 2017), the present study has identified five useful markers of sulfur-fumigation in AB, which include three triterpene saponins (Betavulgarosides II–IV) and two amides (Feruloyl-4-*O*-methyldopamine and Moupinamide) using metabolomic combined with multivariate statistical analysis. We also found that the chemical transformation of three triterpene saponin components declined after sulfur-fumigation, which were positively correlated with OA, while the two amide components that accumulated after sulfur-fumigation were positively correlated with FA. In addition, the degree of the sulfur-fumigation from superficial to deeper tissues were also clarified by UPLC-MS/MS quantitative analysis coupled with PCA/PLS-DA. Combined with the MRL of sulfur dioxide (750 mg/kg) (Kang et al., 2017), the sulfur-fumigation of AB at the ratio of 1:40 within 1 h was sufficient for herbal quality preservation and assurance. Therefore, combined with our published approaches for *Gastrodia Rhizoma*, the sulfur-fumigation protocol has been confirmed to be more reliable for the quality control of sulfur-fumigated herbs.

## Author Contributions

CK performed data investigation and experiments. DZ, LK, SW, CL, LZ, J-YJ, WY, and JL collected and organized the data. L-QH and LG designed the experiments. All authors read and approved the manuscript.

## Conflict of Interest Statement

The authors declare that the research was conducted in the absence of any commercial or financial relationships that could be construed as a potential conflict of interest.

## References

[B1] Bai Y. J. Xu J. D. Kong M. Gao Q. Liu L. F. Li S. L. (2015). Discovery of characteristic chemical markers for inspecting sulfur-fumigated Radix Angelicae Sinensis by ultra-high performance liquid chromatography-quadrupole/time-of-flight mass spectrometry based metabolomics and chemical profiling approach. *Food Res. Int.* 76 387–394. 10.1016/j.foodres.2015.05.055 28455018

[B2] China Pharmacopoeia [Part IV] (2015). *Pharmacopoeia of People’s Republic of China. Part IV.* Beijing: China Medical Science and Technology Press.

[B3] China Pharmacopoeia [Part L] (2015). *Pharmacopoeia of People’s Republic of China. Part L.* Beijing: China Medical Science and Technology Press.

[B4] Duan S. M. Xu J. Bai Y. J. Ding Y. Kong M. Liu H. H. (2016). Sulfur dioxide residue in sulfur-fumigated edible herbs: The fewer, the safer? *Food Chem.* 192 119–124. 10.1016/j.foodchem.2015.07.003 26304328

[B5] European Pharmacopoeia 8.0 (2014). *European Pharmacopoeia 8.0* Vol. 1. Strasbourg: The Directorate for the Quality of Medicines.

[B6] He X. Wang X. Fang J. Chang Y. Ning N. Guo H. (2017). The genus *Achyranthes*: a review on traditional uses, phytochemistry, and pharmacological activities. *J. Ethnopharmacol.* 203 260–278. 10.1016/j.jep.2017.03.035 28347832

[B7] Jiang X. Huang L. F. Zheng S. H. Chen S. L. (2013). Sulfur fumigation, a better or worse choice in preservation of Traditional Chinese Medicine? *Phytomedicine* 20 97–105. 10.1016/j.phymed.2012.09.030 23127540

[B8] Kan W. L. Ma B. Lin G. (2011). Sulfur fumigation processing of traditional Chinese medicinal herbs: beneficial or detrimental? *Front. Pharmacol.* 2:84 10.3389/fphar.2011.00084 PMC3246269 22207851

[B9] Kang C. Zhao D. Zhou T. Liu D.-H. Lv C. Wang S. (2017). A practical protocol for comprehensive evaluation of sulfur-fumigation of *Gastrodia Rhizoma* using metabolome and health risk assessment analysis. *J. Hazard. Mater.* 340 221–230. 10.1016/j.jhazmat.2017.07.003 28715745

[B10] Kong M. Liu H. H. Xu J. Wang C. R. Lu M. Wang X. N. (2014). Quantitative evaluation of Radix Paeoniae Alba sulfur-fumigated with different durations and purchased from herbal markets: simultaneous determination of twelve components belonging to three chemical types by improved high performance liquid chromatography-diode array detector. *J. Pharm. Biomed. Anal.* 98 424–433. 10.1016/j.jpba.2014.06.027 25011060

[B11] Korean Pharmacopoeia XI (2014). *Korean Pharmacopoeia XI.* Osong: Commissioner of the Korea Food and Drug Administration.

[B12] Lai C. J. Zha L. Liu D. H. Kang L. Ma X. Zhan Z. L. (2016). Global profiling and rapid matching of natural products using diagnostic product ion network and in silico analogue database: *Gastrodia elata* as a case study. *J. Chromatogr. A* 1456 187–195. 10.1016/j.chroma.2016.06.013 27318507

[B13] Li J. X. Hareyama T. Tezuka Y. Zhang Y. Miyahara T. Kadota S. (2005). Five new oleanolic acid glycosides from *Achyranthes bidentata* with inhibitory activity on osteoclast formation. *Planta Med.* 71 673–679. 10.1055/s-2005-871275 16041655

[B14] Li R. J. Kou X. J. Tian J. J. Meng Z. Q. Cai Z. W. Cheng F. Q. (2014). Effect of sulfur dioxide on inflammatory and immune regulation in asthmatic rats. *Chemosphere* 112 296–304. 10.1016/j.chemosphere.2014.04.065 25048919

[B15] Li S. L. Shen H. Zhu L. Y. Xu J. Jia X. B. Zhang H. M. (2012). Ultra-high-performance liquid chromatography-quadrupole/time of flight mass spectrometry based chemical profiling approach to rapidly reveal chemical transformation of sulfur-fumigated medicinal herbs, a case study on white ginseng. *J. Chromatogr. A* 1231 31–45. 10.1016/j.chroma.2012.01.083 22360913

[B16] Li X. Y. Long F. Xu J. D. Shen H. Kong M. Zhu H. (2017). Paeonifiorin sulfonate as a characteristic marker for specifically inspecting Chinese patent medicine Liu-Wei-Di-Huang-Wan contained sulfur-fumigated Moutan Cortex. *J. Pharm. Biomed. Anal.* 138 283–288. 10.1016/j.jpba.2017.02.029 28231532

[B17] Li X.-Y. Xu J.-D. Xu J. Kong M. Zhou S.-S. Mao Q. (2016). UPLC-QTOF-MS based metabolomics coupled with the diagnostic ion exploration strategy for rapidly evaluating sulfur-fumigation caused holistic quality variation in medicinal herbs, Moutan Cortex as an example. *Anal. Methods* 8 1034–1043. 10.1039/C5AY01404B

[B18] Li Z. Wang Y. Ouyang H. Lu Y. Qiu Y. Feng Y. (2015). A novel dereplication strategy for the identification of two new trace compounds in the extract of *Gastrodia elata* using UHPLC/Q-TOF-MS/MS. *J. Chromatogr. B* 988 45–52. 10.1016/j.jchromb.2015.02.020 25746751

[B19] Lin L. F. Lin H. M. Zhang M. Ni B. R. Yin X. B. Qu C. H. (2015). A novel method to analyze hepatotoxic components in *Polygonum multiflorum* using ultra-performance liquid chromatography-quadrupole time-of-flight mass spectrometry. *J. Hazard. Mater.* 299 249–259. 10.1016/j.jhazmat.2015.06.014 26135484

[B20] Liu Y. Yang F. Pu H. Su J. Liu Z. Dhiloo K. H. (2016). The Sublethal Effects of beta-Ecdysterone, a Highly Active Compound from *Achyranthes bidentata* blume, on grape phylloxera, *Daktulosphaira vitifoliae* Fitch. *PLoS One* 11:e0165860. 10.1371/journal.pone.0165860 27824901 PMC5100988

[B21] Ma B. Kan W. L. Zhu H. Li S. L. Lin G. (2017). Sulfur fumigation reducing systemic exposure of ginsenosides and weakening immunomodulatory activity of ginseng. *J. Ethnopharmacol.* 195 222–230. 10.1016/j.jep.2016.11.023 27856301

[B22] Ma X.-Q. Man Leung A. K. Chan C. L. Su T. Li W.-D. Li S.-M. (2014). UHPLC UHD Q-TOF MS/MS analysis of the impact of sulfur fumigation on the chemical profile of Codonopsis Radix (Dangshen). *Analyst* 139 505–516. 10.1039/c3an01561k 24286102

[B23] Mitaine-Offer A. C. Marouf A. Hanquet B. Birlirakis N. Lacaille-Dubois M. A. (2001a). Two triterpene saponins from *Achyranthes bidentata*. *Chem. Pharm. Bull.* 49 1492–1494. 10.1248/cpb.49.1492 11724247

[B24] Mitaine-Offer A.-C. Marouf A. Pizza C. Khanh T. C. Chauffert B. Lacaille-Dubois M.-A. (2001b). Bidentatoside I, a new triterpene saponin from *Achyranthes bidentata*. *J. Nat. Prod.* 64 243–245. 11430011 10.1021/np000464a

[B25] Pei K. Cai H. Liu X. Tu S. Cao G. Li H. (2015). Evaluation of the influence of sulfur fumigation on the pharmacokinetics of four active ingredients in Si Wu Tang. *J. Sep. Sci.* 38 25–33. 10.1002/jssc.201400874 25354295

[B26] Shen S. Wang Q. Li Y. B. (2011). Studies on chemical constituents and pharmaceutics activity of *Achyranthes bidentata* Bl. *Strait Pharm. J.* 23 1–6.

[B27] Sun X. Cui X. B. Wen H. M. Shan C. X. Wang X. Z. Kang A. (2017). Influence of sulfur fumigation on the chemical profiles of *Atractylodes macrocephala* Koidz. evaluated by UFLC-QTOF-MS combined with multivariate statistical analysis. *J. Pharm. Biomed. Anal.* 141 19–31. 10.1016/j.jpba.2017.03.003 28414971

[B28] United States Pharmacopoeia (2014). *United States Pharmacopoeia 38.* Rockville, MD: United States Pharmacopeial Convention Inc.

[B29] Vetrichelvan T. Jagadeesan M. (2002). Effect of alcoholic extract of *Achyranthes bidentata* blume on acute and sub acute inflammation. *Indian J. Pharmacol.* 34 115–118.

[B30] Yan S.-X. Zhang L. Mao R.-L. Zhu H. Li Y. (2016). Assessment of genetic diversity and population differentiation of *Achyranthes bidentata* (Amaranthaceae) in Dao Di and its surrounding region based on microsatellite markers. *Biochem. Syst. Ecol.* 69 27–32. 10.1016/j.bse.2016.08.008

[B31] Yan Y. Zhang Q. Feng F. (2016). HPLC-TOF-MS and HPLC-MS/MS combined with multivariate analysis for the characterization and discrimination of phenolic profiles in nonfumigated and sulfur-fumigated rhubarb. *J. Sep. Sci.* 39 2667–2677. 10.1002/jssc.201501382 27173451

[B32] Yang L. Jiang H. Yan M. L. Xing X. D. Zhang Y. Y. Wei N. (2017). A new phytoecdysteroid from the roots of *Achyranthes bidentata* Bl. *Nat. Prod. Res.* 31 1073–1079. 10.1080/14786419.2016.1272114 28033715

[B33] Yuan Y. Shen H. Yao J. Hu N. Ding F. Gu X. (2010). The protective effects of *Achyranthes bidentata* polypeptides in an experimental model of mouse sciatic nerve crush injury. *Brain Res. Bull.* 81 25–32. 10.1016/j.brainresbull.2009.07.013 19646511

