# Supplementary material for: Elucidation of Characteristic Sulfur-Fumigated Markers and Chemical Transformation Mechanism for Quality Control of Achyranthes bidentate Blume Using Metabolome and Sulfur Dioxide Residue Analysis
Source: Front Plant Sci. 2018 Jun 12;9:790. doi: 10.3389/fpls.2018.00790 (PMC6007317; doi:10.3389/fpls.2018.00790)
Supplement: Supplementary file 1 [file Data_Sheet_1.docx]

**Elucidation of characteristic sulfur-fumigated markers and chemical transformation mechanism for quality control of *Achyranthes bidentate* Blume using** **metabolome and sulfur dioxide residue analysis**

Chuanzhi Kang ^1^, Dan Zhao ^2^, Liping Kang ^1^, Sheng Wang ^1^, Chaogeng Lv ^1^, Li Zhou ^1^, Jing-Yi Jiang ^1^, Wanzhen Yang ^1^, Jiaxing Li ^1^, Lu-Qi Huang ^1, *^, Lanping Guo ^1, *^

a National Resource Center for Chinese Materia Medica, China Academy of Chinese Medical Sciences, State Key Laboratory Breeding Base of Dao-di Herbs, Beijing, 100700, PR China

b Guiyang University of Chinese Medicine, Guiyang 550025, PR China

^*^Corresponding authors.

E-mail addresses: huangluqi01@126.com (L-Q. Huang), glp01@126.com (L. Guo).

**Supplementary data**


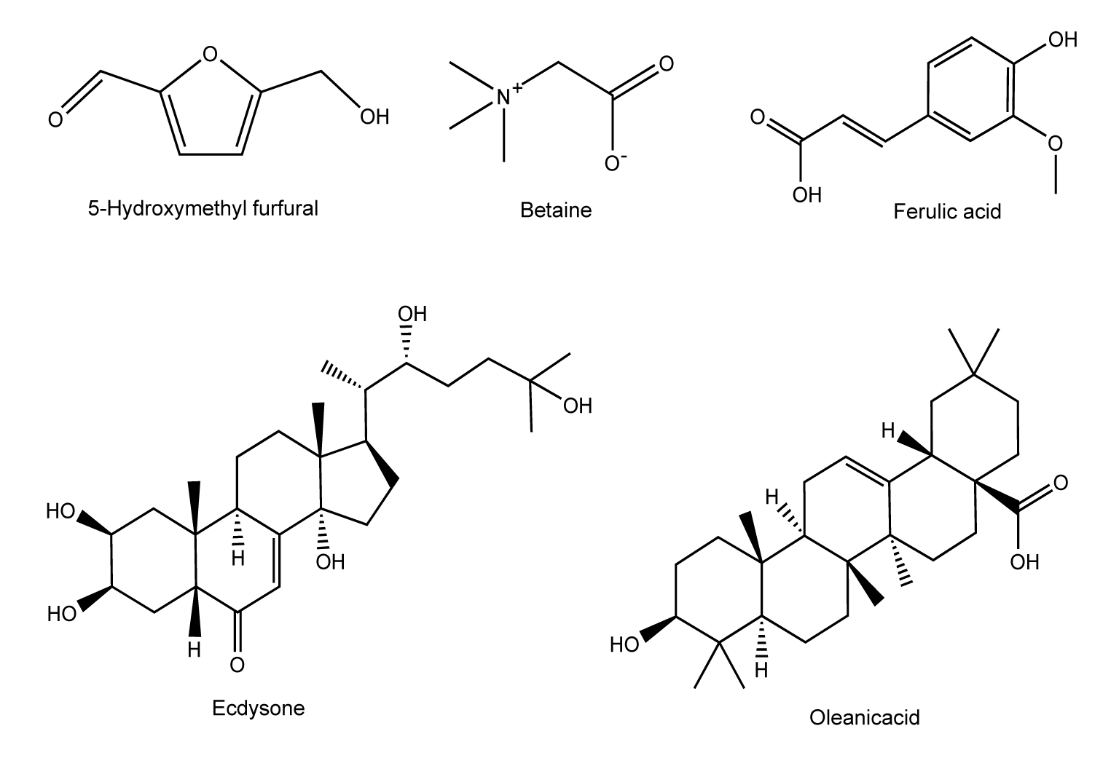


**FIGURE S1**. Chemical structures of five reference compounds in AB.


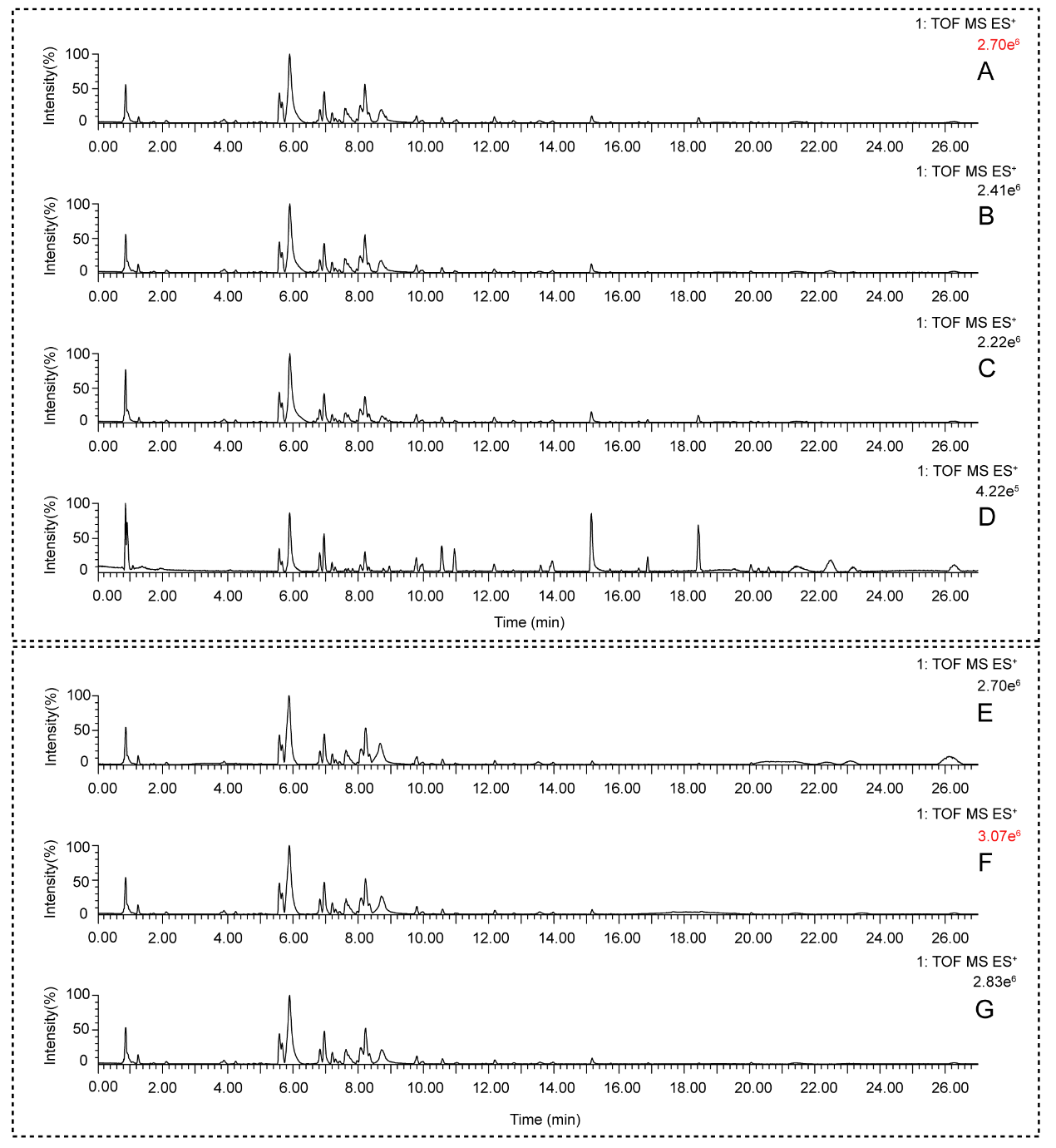


**FIGURE S2.** Optimized extraction conditions selected from four extraction solvents and three extraction time. A: 50% methanol; B: 80% methanol; C: methanol; D: ethanol; E: 30min; F: 60min; G: 90min.

**
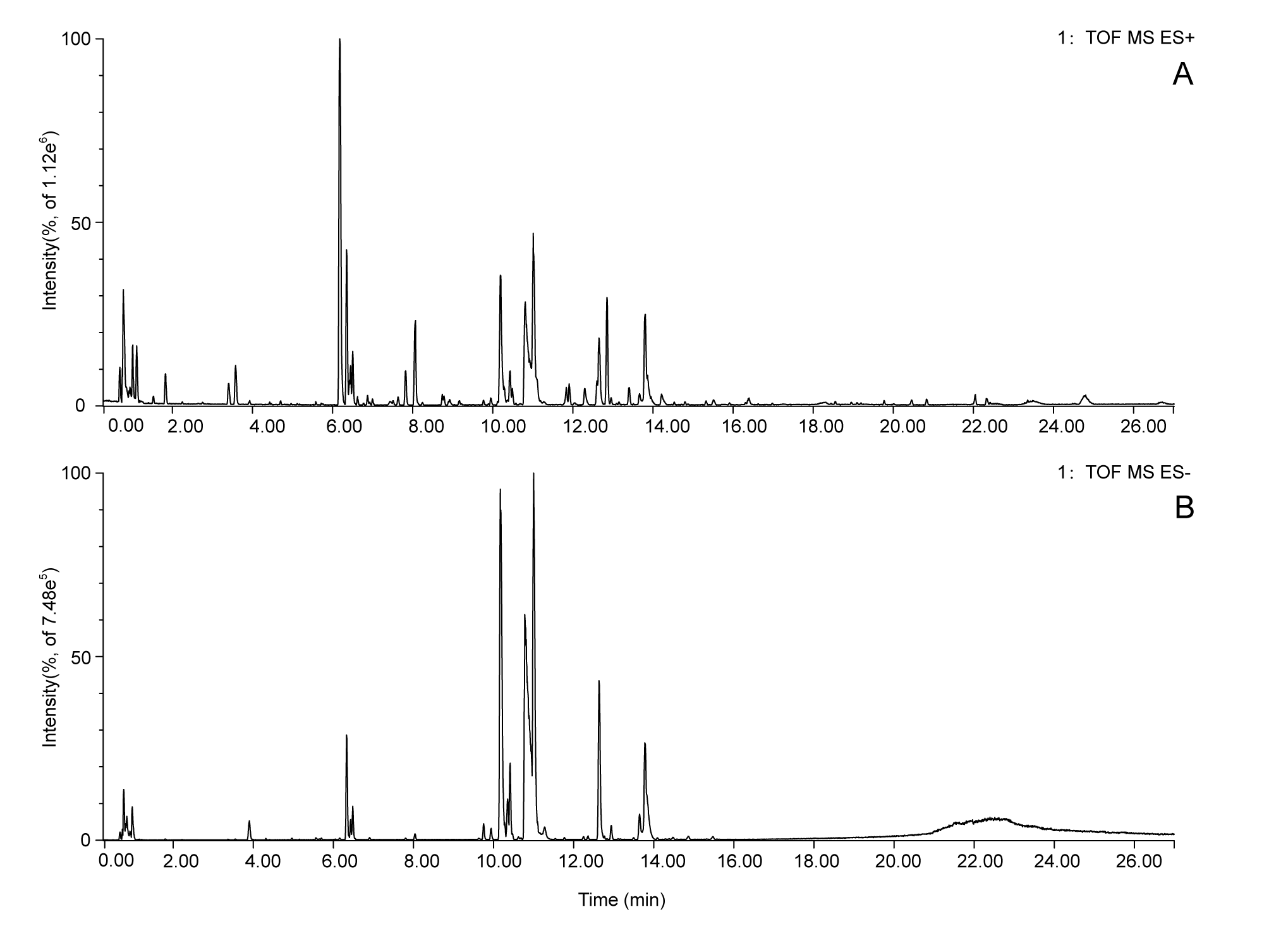
**

**FIGURE S3** Base peak ions (BPI) of AB extraction in positive mode (A) and negative mode (B).

**
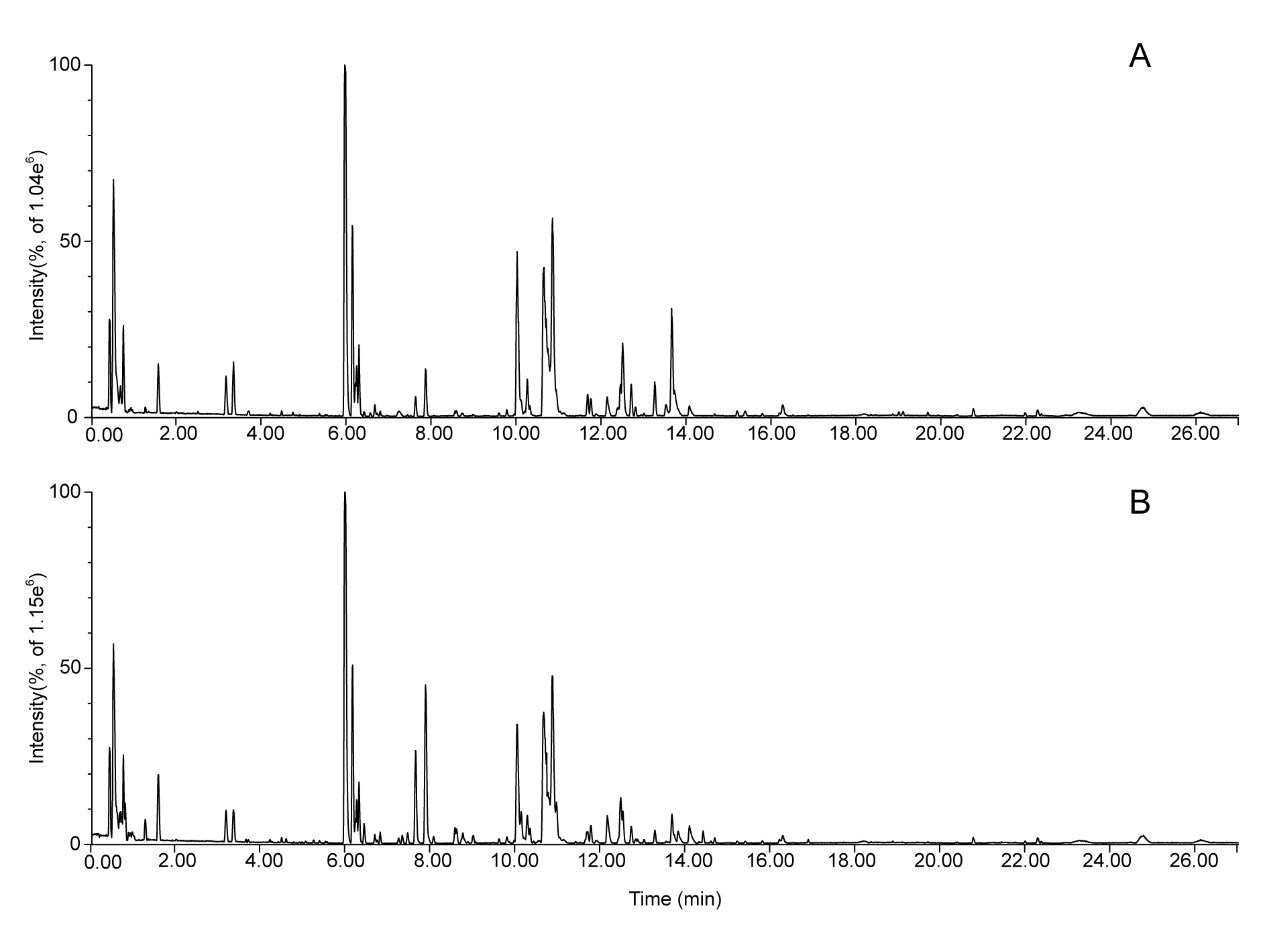
**

**FIGURE S4** Total ion chromatograms of AB extraction for non-fumigated (A) and sulfur-fumigated (B) in positive mode.


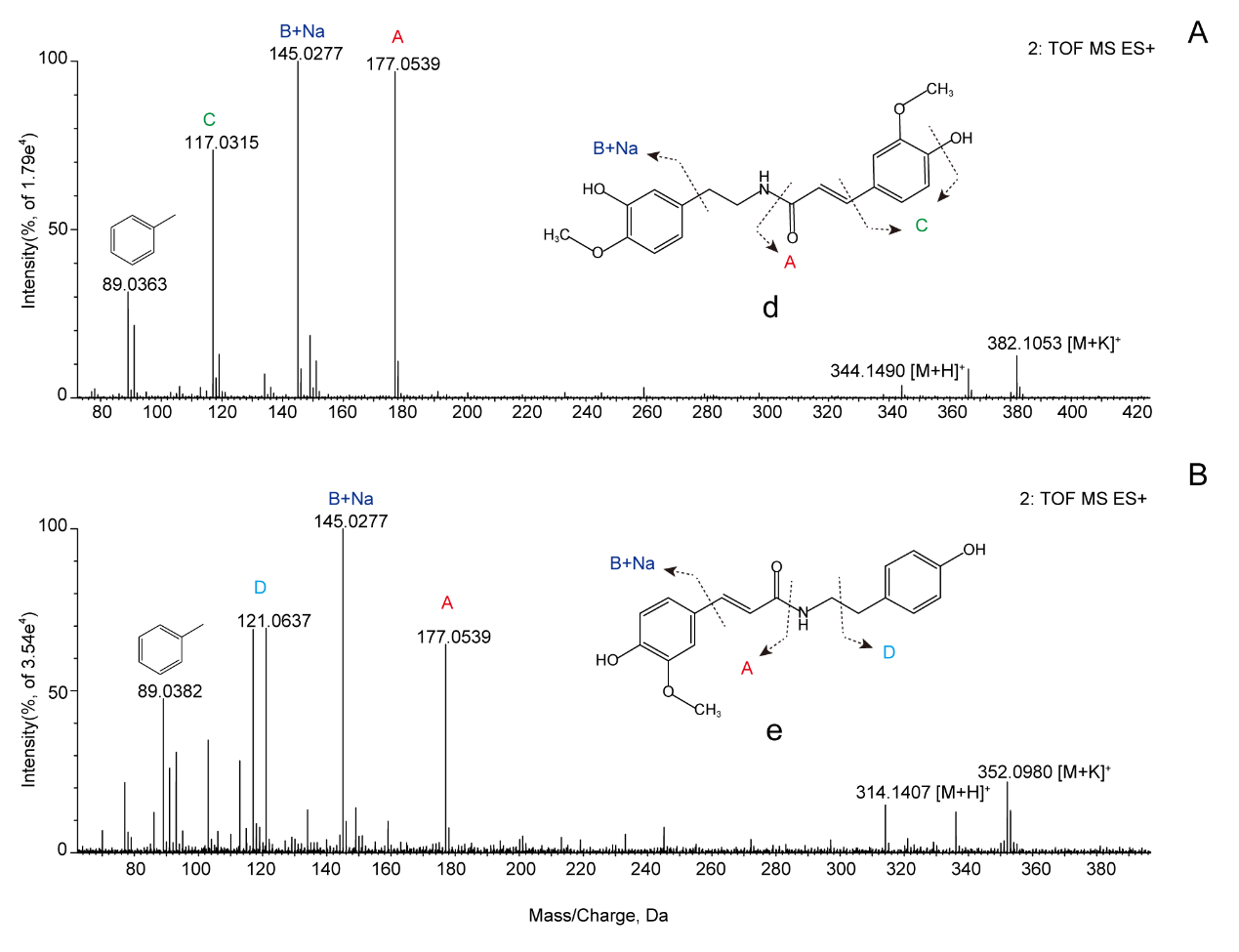


**FIGURE S5** The positive MS/MS spectrum of characteristic chemical markers. A: marker d (Feruloyl-4-O-methyldopamine); B: marker e (Moupinamide).


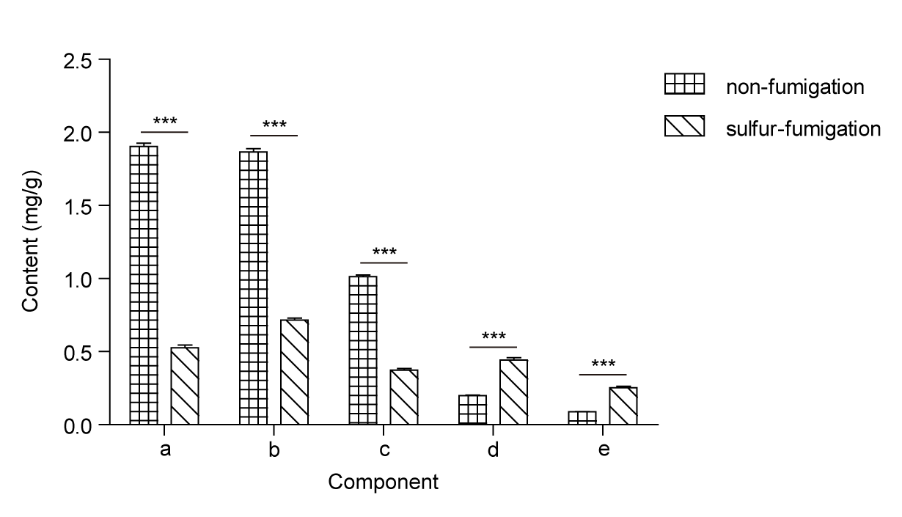


**FIGURE S6** Relative content of five characteristic markers in non-fumigated and sulfur-fumigated AB (*n*=6, ^***^*p*<0.001).


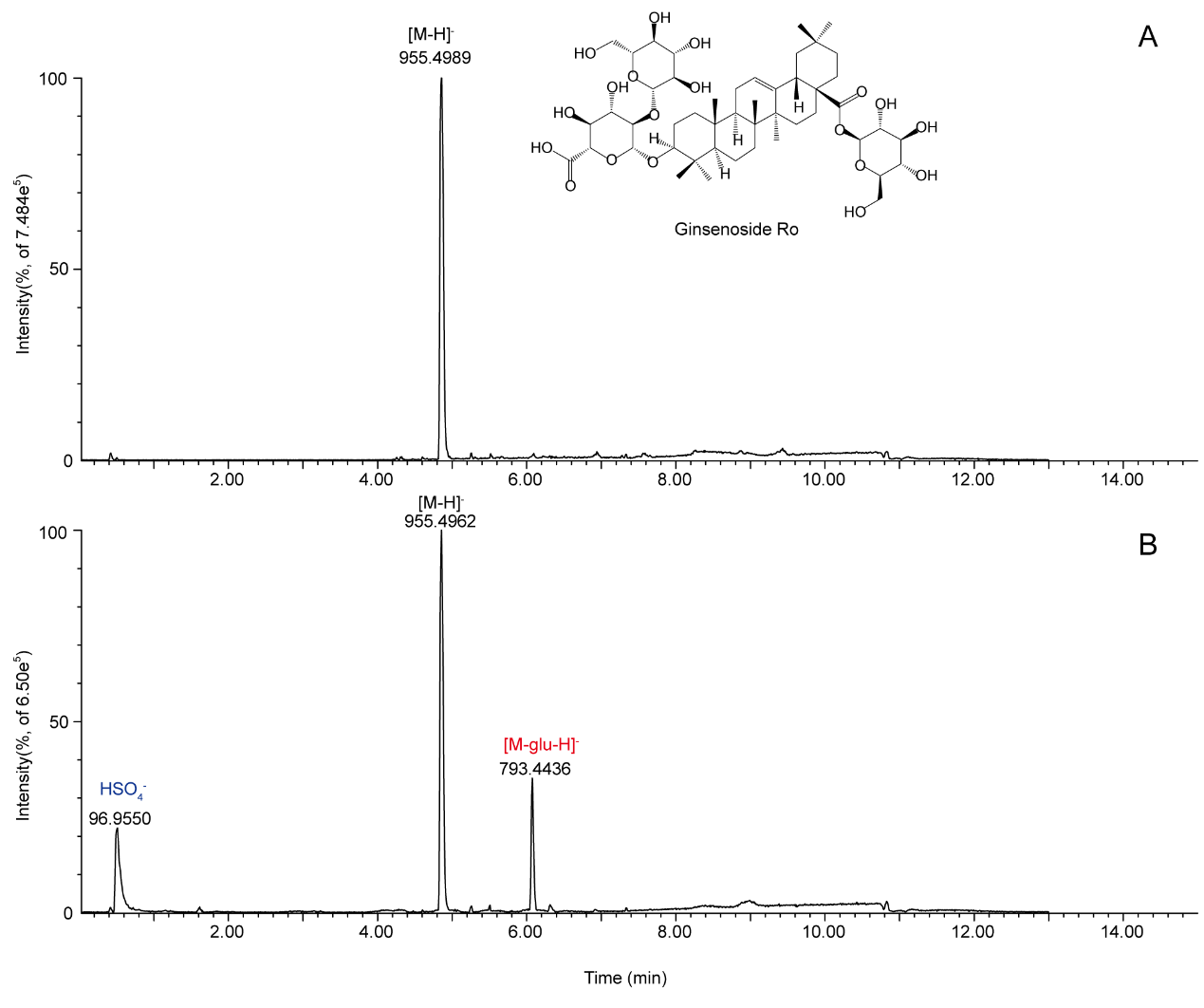


**FIGURE S7** Total ion chromatograms of ginsenoside Ro under sulfur fumigation. (A): ginsenoside Ro standard without sulfur fumigation; (B): hydrolytic reactions of ginsenoside Ro under sulfur fumigation.


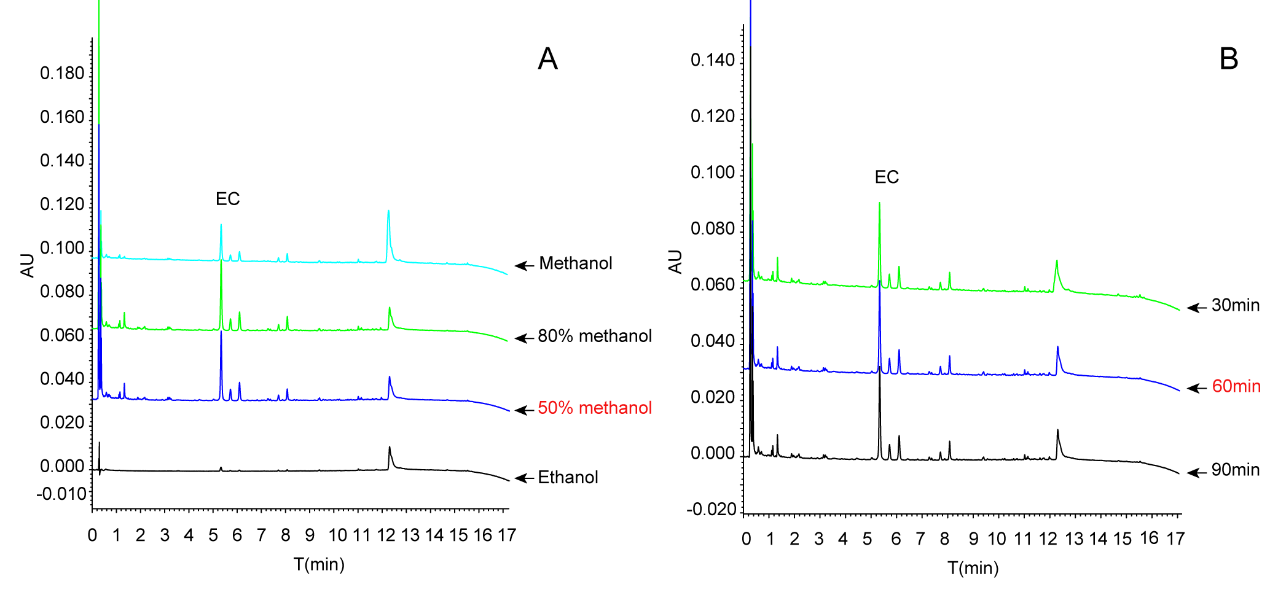


**FIGURE S8** Typical chromatograms for quantitative analysis of AB. (A): four extraction solvent; (B): three extraction time


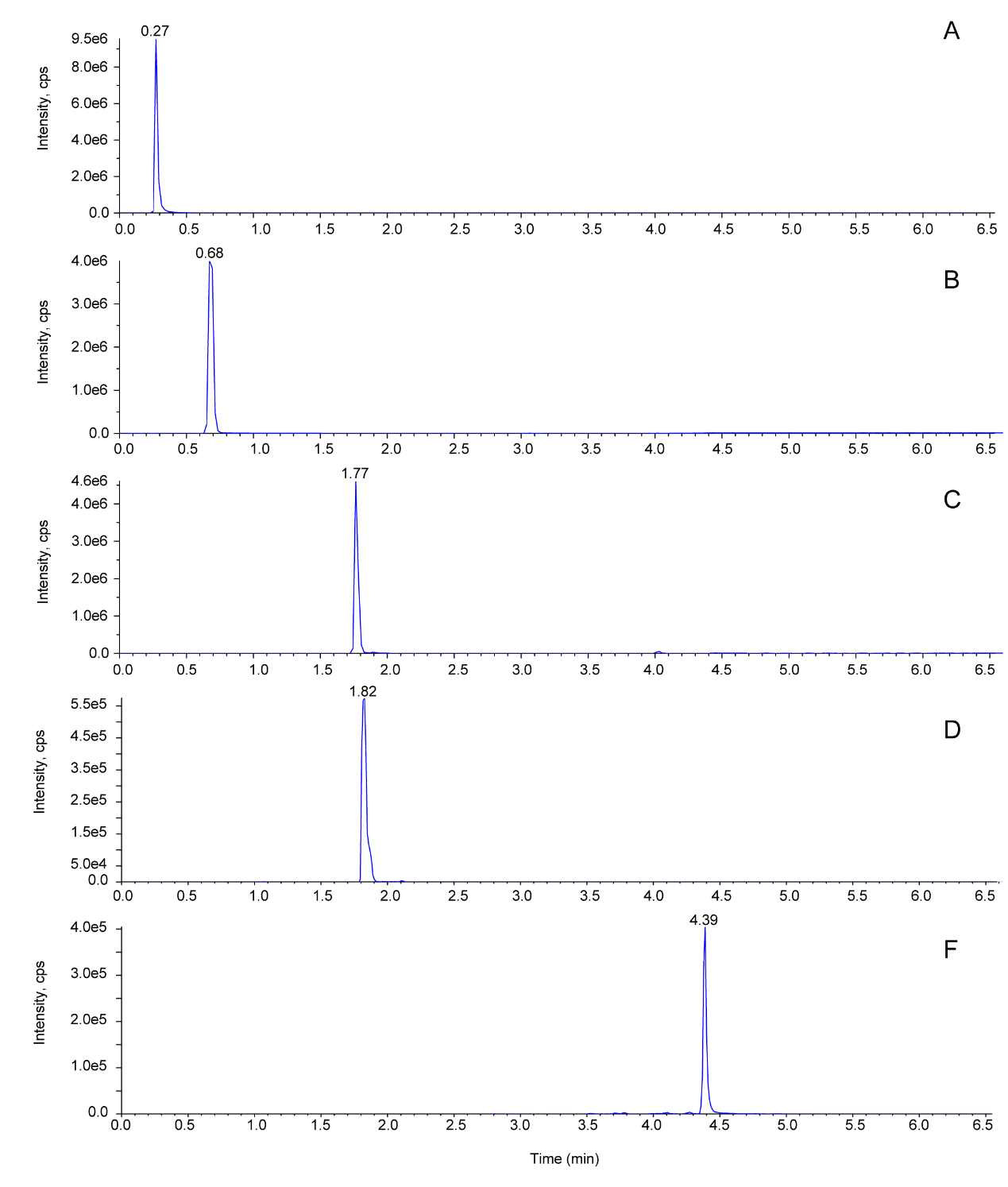


**FIGURE S9** MRM chromatogram of five analytes in AB by UPLC–MS/MS. A: BE; B: 5-HF; C: FA; D: EC; F: OA.

**TABLE S1**. LC-MS date of five compounds in AB.

| Compounds | Rt  /min | MRM^b^ | | | | |
| --- | --- | --- | --- | --- | --- | --- |
|  |  | Q1/Q3 ion^a^ (*m/z*) | DP/V | CE/eV | CXP/V | EP/V |
| EC | 1.82 | 479.2/318.9 | -79 | -34 | -14 | -15 |
| OA | 4.39 | 455.3/407.3 | -10 | -50 | -11 | -15 |
| BE | 0.27 | 118.2/57.9 | 11 | 34 | 6 | 15 |
| FA | 1.77 | 195.2/177.0 | 3 | 13 | 6 | 15 |
| 5-HF | 0.68 | 127.1/109.0 | 31 | 13 | 13 | 15 |
| Ion spray voltage (IS) /V | -4500/5500 | | | | | |
| Ion source (GS1) setting/Pis | 55 | | | | | |
| Ion source (GS2) setting/Pis | 55 | | | | | |

^a^ Q1: precursor ion selected in Q1; Q3: product ion selected in Q3; ^b^ DP: declustering potential; CE: collision energy; EP: entrance potential; CXP: collision cell exit potential.

**TABLE S2**. Recovery of five compounds in AB (*n*=3).

| Analyte | Initial amount (μg) | Added amount (μg) | Total recovered amount (μg) | Recovery (%) | RSD (%) |
| --- | --- | --- | --- | --- | --- |
| FA | 0.0208 | 0.0166 | 0.0382 | 103.4190±3.3503 | 3.24 |
|  | 0.0208 | 0.0207 | 0.0419 | 101.3947±0.8150 | 0.80 |
|  | 0.0204 | 0.0248 | 0.0453 | 98.7789±1.7760 | 1.80 |
| EC | 75.7482 | 60.0816 | 132.3293 | 96.4625±1.9873 | 2.06 |
|  | 75.6028 | 75.1020 | 144.1791 | 93.0055±2.4239 | 2.61 |
|  | 74.0035 | 90.1224 | 161.2800 | 96.7253±2.4962 | 2.58 |
| 5-HF | 0.0261 | 0.0206 | 0.0455 | 94.4484±1.1544 | 1.22 |
|  | 0.0260 | 0.0258 | 0.0505 | 97.0064±3.2164 | 2.46 |
|  | 0.0255 | 0.0310 | 0.0582 | 102.2688±3.2164 | 3.15 |
| BE | 329.1886 | 261.1044 | 591.0767 | 98.3215±2.3932 | 2.43 |
|  | 328.5568 | 326.3805 | 651.5690 | 99.8439±2.9215 | 2.93 |
|  | 321.6066 | 391.6566 | 727.1171 | 104.9982±1.3311 | 1.27 |
| OA | 413.2520 | 327.7814 | 735.4690 | 97.9451±2.1022 | 2.15 |
|  | 412.4588 | 409.7267 | 846.8389 | 105.3923±0.9274 | 0.88 |
|  | 403.7337 | 491.6720 | 906.1941 | 103.4355±2.2977 | 2.22 |

**TABLE S3** Content of the five major compounds in the AB samples within 24 h sulfur-fumigation (*n*=3，‾x±s)

| Sulfur dose | Sulfur-fumigation time(h) | FA  /(mg/g) | EC  /(mg/g) | 5-HF  /(mg/g) | BE  /(mg/g) | OA  /(mg/g) |
| --- | --- | --- | --- | --- | --- | --- |
| Blank | 0 | 0.0004±0.0000 ^b^ | 1.4421±0.0724 | 0.0005±0.0000 | 6.4671±0.1693 | 7.4075±0.5506 |
|  | 1 | 0.0003±0.0000 | 1.3800±0.0236 | 0.0006±0.0000 | 6.3134±0.0877 | 7.0556±0.3352 |
|  | 2 | 0.0003±0.0000 | 1.4455±0.0608 | 0.0005±0.0000 | 6.3034±0.0600 | 7.1022±0.3399 |
|  | 4 | 0.0005±0.0000 | 1.3128±0.0776 | 0.0006±0.0000 | 6.3263±0.1498 | 7.2072±0.2155 |
|  | 8 | 0.0004±0.0000 | 1.3972±0.0854 | 0.0006±0.0000 | 6.4605±0.1789 | 7.2097±0.3160 |
|  | 12 | 0.0004±0.0000 | 1.3167±0.0904 | 0.0006±0.0000 | 6.3836±0.1755 | 7.0329±0.2003 |
|  | 24 | 0.0004±0.0001 | 1.3927±0.0636 | 0.0006±0.0000 | 6.4436±0.1844 | 7.3559±0.5129 |
| 1:20 ^a^ | 0 | 0.0007±0.0000 | 1.5242±0.0422 | 0.0010±0.0000 | 5.8068±0.1017 | 8.9145±0.3441 |
|  | 1 | 0.0001±0.0000 | 1.3237±0.0817 | 0.0039±0.0002 | 5.4571±0.1353 | 5.4604±0.0367 |
|  | 2 | 0.0028±0.0001 | 1.3695±0.0705 | 0.0054±0.0002 | 5.5750±0.1074 | 6.5795±0.3707 |
|  | 4 | 0.0027±0.0001 | 1.3060±0.0744 | 0.0055±0.0001 | 5.4229±0.0988 | 4.0628±0.1717 |
|  | 8 | 0.0022±0.0001 | 1.3029±0.0466 | 0.0064±0.0003 | 5.3063±0.2110 | 4.0841±0.1785 |
|  | 12 | 0.0021±0.0001 | 1.2946±0.0372 | 0.0086±0.0005 | 5.2265±0.1439 | 4.0782±0.0933 |
|  | 24 | 0.0020±0.0001 | 1.3053±0.0607 | 0.0174±0.0008 | 5.2078±0.1935 | 4.0567±0.1504 |
| 1:40 | 0 | 0.0004±0.0001 | 1.5753±0.0244 | 0.0016±0.0001 | 5.1425±0.1329 | 8.8958±0.4947 |
|  | 1 | 0.0023±0.0001 | 1.3945±0.0632 | 0.0039±0.0002 | 5.0030±0.2322 | 7.0088±0.3648 |
|  | 2 | 0.0024±0.0000 | 1.4723±0.0564 | 0.0050±0.0002 | 4.8794±0.1956 | 8.1324±0.4846 |
|  | 4 | 0.0021±0.0001 | 1.3917±0.0886 | 0.0054±0.0003 | 4.8252±0.1149 | 4.3604±0.2264 |
|  | 8 | 0.0021±0.0001 | 1.3921±0.0375 | 0.0114±0.0005 | 4.7819±0.1917 | 4.3252±0.2112 |
|  | 12 | 0.0023±0.0001 | 1.4055±0.0500 | 0.0118±0.0007 | 4.8299±0.1566 | 4.3478±0.1331 |
|  | 24 | 0.0020±0.0000 | 1.3954±0.0415 | 0.0176±0.0008 | 4.7996±0.1843 | 4.2805±0.1646 |
| 1:80 | 0 | 0.0006±0.0000 | 1.5517±0.0504 | 0.0017±0.0001 | 4.2355±0.1843 | 9.2994±0.3756 |
|  | 1 | 0.0001±0.0000 | 1.5493±0.0367 | 0.0029±0.0001 | 4.1339±0.0948 | 8.9488±0.1642 |
|  | 2 | 0.0002±0.0000 | 1.4677±0.0690 | 0.0032±0.0002 | 4.1610±0.0948 | 7.1165±0.3897 |
|  | 4 | 0.0022±0.0001 | 1.4651±0.1025 | 0.0045±0.0001 | 4.0406±0.1420 | 6.8222±0.3084 |
|  | 8 | 0.0018±0.0001 | 1.4230±0.0833 | 0.0052±0.0003 | 4.0548±0.0336 | 6.4791±0.3041 |
|  | 12 | 0.0018±0.0001 | 1.4295±0.0654 | 0.0086±0.0005 | 4.0427±0.0762 | 5.0811±0.2354 |
|  | 24 | 0.0017±0.0001 | 1.4022±0.0677 | 0.0116±0.0005 | 4.0243±0.0745 | 5.1450±0.1416 |

^a^ The weight ratio of sulfur and herbal material.

^b^ Values are expressed as mean±standard deviations(*n*=3) in mg/g
